# Modeling the neuro-mechanics of human balance when recovering from a fall: a continuous-time approach

**DOI:** 10.1186/s12938-020-00811-1

**Published:** 2020-08-31

**Authors:** Angel Cerda-Lugo, Alejandro González, Antonio Cardenas, Davide Piovesan

**Affiliations:** 1 Faculty of Engineering, Universidad Autónoma de San Luis Potosí, San Luis Potosí, Mexico; 2 Faculty of Engineering, CONACyT-Universidad Autónoma de San Luis Potosí, San Luis Potosí, Mexico; 3 Biomedical, Industrial and Systems Engineering Department, Gannon University, Erie, PA USA

**Keywords:** Human balance, Parameter estimation, Dynamic model

## Abstract

**Background:**

Balance control deteriorates with age and nearly 30% of the elderly population in the United States reports stability problems. Postural stability is an integral task to daily living reliant upon the control of the ankle and hip. To this end, the estimation of joint parameters can be a useful tool when analyzing compensatory actions aimed at maintaining postural stability.

**Methods:**

Using an analytical approach, this study expands on previous work and analyzes a two degrees of freedom human model. The first two modes of vibration of the system are represented by the neuro-mechanical parameters of a second-order, time-varying Kelvin–Voigt model actuated at the ankle and hip. The model is tested using a custom double inverted pendulum and healthy volunteers who were subjected to a positional step-like perturbation during quiet standing. An in silico sensitivity analysis of the influence of inertial parameters was also performed.

**Results:**

The proposed method is able to correctly identify the time-varying visco-elastic parameters of of a double inverted pendulum. We show that that the parameter estimation method can be applied to standing humans. These results appear to identify a subject-independent strategy to control quiet standing that combines both the modulation of stiffness, and the use of an intermittent control.

**Conclusions:**

This paper presents the analysis of the non-linear system of differential equations representing the control of lumped muscle–tendon units. It utilizes motion capture measurements to obtain the estimates of the system’s control parameters by constructing a simple time-dependent regressor for estimating the time-varying parameters of the control with a single perturbation. This work is a step forward into the understanding of the neuro-mechanical control parameters of human recovering from a fall. In previous literature, the analysis is either restricted to the first vibrational mode of an inverted-pendulum model or assumed to be time-invariant. The proposed method allows for the analysis of hip related movement for stability control and highlights the importance of core training.

## Background

In the United States, approximately 30% of older adults, suffer from different types of balance impairments [1]. This particularly affects the elderly. Death in the elderly population can often be traced back to a fall, where the individual has lost balance and broken a leg, usually at the hip. Sudden immobilization due to fracture has adverse effects in an individual’s health. Mainly a reduction in activity levels [2] which could, in turn, lead to diminished bone mass [3] and cardiovascular performance [4]. Thus, the need to prevent falls which starts with understanding of the margin of stability in upright balance and how the modulation of joint stiffness impacts said margin.

Our previous work has focused on modeling the quiet standing and recovery from fall using a single degree of freedom inverted pendulum [5]. We have proposed two different measuring setups for the identification of the ankle stiffness [6–8]. This variable has been considered as a figure of merit to assess the balance stability of an individual [9]. On the other hand, using higher complexity models could give the opportunity to highlight different control strategies that involve the co-ordinated control of multiple degrees of freedom [10].

Note that, henceforth, the term stiffness is extended from the usual definition of a linear time-invariant coefficient that correlates joint displacement with joint-generated torque. We will assume that this coefficient is time-varying and is representative of the neuro-mechanical control of the joint. Stiffness is in charge of either storing elastic energy (e.g., in the tendon when the stiffness is positive), or injecting energy into the system (e.g., from active work produced by the muscle via segmental reflexes) in which case the stiffness can be considered negative.

In the present work, we have used the *hold and release* paradigm proposed by Bortolami et al. [11], combined with a time-based system-identification technique to determine the time-varying stiffness at the hip and ankle that allows individuals to recover from a fall.

Human balance is an intrinsically unstable task. The research on this topic has been extensive, due primarily to the deleterious consequence that a fall can have, particularly in the elderly. Differences in modeling human quiet standing can be found in: (i) the number of equivalent body segments utilized to model the human, (ii) the mechanical model of the joint, and (iii) the estimation method.

### Number of body segments

The number of segments modeled to represent human quiet-standing dictates the number of (differential) equations utilized to describe the physical system. The field of structural engineering provides an excellent background for this representation, where each human segment (i.e., shank, leg, torso, head) can be equivalent to a floor of a building (see for example [12]). Structural dynamics points out that the mechanical power of the oscillation is distributed within basic functions at different frequencies. The lower frequency modes have larger amplitudes and describe a large amount of variation in the position of the center of mass. The first mode of vibration often accounts for 80% of the variance of the center of mass displacement of the whole system [11]. Thus, a simple model of a single inverted pendulum can be a representation of the first mode of vibration of a more complex system. A single inverted pendulum can provide a simple first principle model of the control of human balance by controlling the torque vs. angular displacement at the ankle [13]. This model can provide a framework in line with the equilibrium point hypothesis [14, 15], where the control scheme is simple, and it exploits the mechanical properties of the muscles for a correction without delay. These models, however, cannot account for many practical problems such as their high energy consumption, where continuous muscle co-contraction requires high metabolic energy [10, 16]. Furthermore, the single oscillatory mode hypothesis, where only the ankle is controlled, was challenged by the experimental evidence that the movement at the hip during quiet standing is not negligible [17–19]. In particular, such hip movements were found to be phase-locked with those of the ankle, suggesting that higher modes of vibrations appear to be essential to understand how balance is maintained.

### Mechanical model of the joint

Having concluded that a single-mode oscillation provides an incomplete picture of the control scheme, we would need now to assume that at least two degrees of freedom are controlled at the same time. These calculations demonstrate that the double-link inverted-pendulum model provides a more conservative estimate of minimum stiffness [20]. Furthermore, we can infer the nature of the control by monitoring how the control parameters at each joint vary in time. As a first approximation, assuming a linear relationship between the torque and the angular displacement of each joint would reduce the complexity of the model. This is equivalent to expecting a constant stiffness for each joint. Note that, the invariability of the stiffness does not imply that the system is passive, as a continuous level of muscles’ co-contraction is required to achieve a critical stiffness able to stabilize the system with a reasonable margin of stability [21]. It has been demonstrated that the magnitude of ankle intrinsic stiffness is insufficient to maintain stability [10, 16], and therefore, a neural modulation is required, which would, in turn, modulate the mechanics of the joint in time [22]. Therefore, it is essential to assume a time-varying model to represent the neuromechanics of the joint.

### Estimation method

Many estimations of ankle neuro-mechanical parameters during quiet standing and walking are obtained via a regression. Regressive techniques are accurate but have two main drawbacks. They can only provide an average value of the parameter within a short time window where the parameter is assumed constant, and require the assumption of ergodicity, i.e., that the parameter remains the same over the number of repetitions necessary to obtain the estimate. Examples of this technique can be found in [16, 23, 24], which consider the subject as standing on a controllable mechatronic platform mimicking experimental conditions similar to everyday activities. To estimate time-varying neuro-mechanical parameters for the ankle non-parametric frequency-based approaches have also been used [25–27]. The advantage of these techniques is that they are model free. On the other hand, they require a large amount of data, and the experiments are performed in conditions that do not mimic everyday activity, where the subject is seated in a chair, and the foot is rotated about the ankle by a mechatronic system. Regression and frequency-based techniques have some common challenges. They use multiple external perturbations to observe a change in the ankle neuro-mechanical parameters and cannot obtain a reliable estimate during a single trial. Furthermore, the very nature of the techniques does not allow for estimating the parameters of multiple joints (i.e., ankle and hip) at the same time. The application of external perturbations to the whole body to elicit change in multiple joint neuro-muscular parameters is not trivial. The disturbance requires enough power to excite the higher mode of vibrations associated with multiple joints. The power required to activate higher modes of vibrations is proportional to the square of their amplitude and the cube of their natural frequency [28]. Thus, as more segments are added to the model, the mechanical power required to elicit an observable change in joint kinematics increases dramatically. Remarkably, the *hold and release* paradigm [11] is able to use the internal dynamics of the body as a perturbation allowing for enough power to elicit measurable changes in multiple joints. The technique used to estimate the neuro-mechanical parameters, in this case, is based on the estimation of a time series. An extended Kalman filter is an ideal tool for the estimation. This technique is unique as it can provide a time-varying estimate of multiple joint neuro-mechanical parameters within one single trial.

The final objective of this project is to find which control strategies individuals can utilize to prevent falling, and how stiffness and damping at specific joints are modulated. For this specific work, we are using the experimental paradigm of Bortolami et al. [11] that encompasses the first two modes of vibration of a standing human. The technique proposed in this paper will bring us closer to the practical goal of creating an inexpensive setup to assess the risk of fall in elderly individuals. This study presents the non-linear equations of motion and how they are used for the construction of a Kalman filter (KF). This work is different from the previous literature [29] as it represents both the behavior at the hip and ankle, estimating a time-varying stiffness with only one perturbation. After a thorough validation of the technique, we present a set of direct measurements on human subjects where a consistent and repeatable control strategy can be highlighted. Specifically, we observed that the hip stiffness is maintained roughly constant and the ankle stiffness actively changes sign so to inject or dissipate energy into the system thus simplifying the control strategy. Finally, we show by simulation that this is a valid balancing strategy and can result in attaining an upright posture.

## Results

**Fig. 1:**
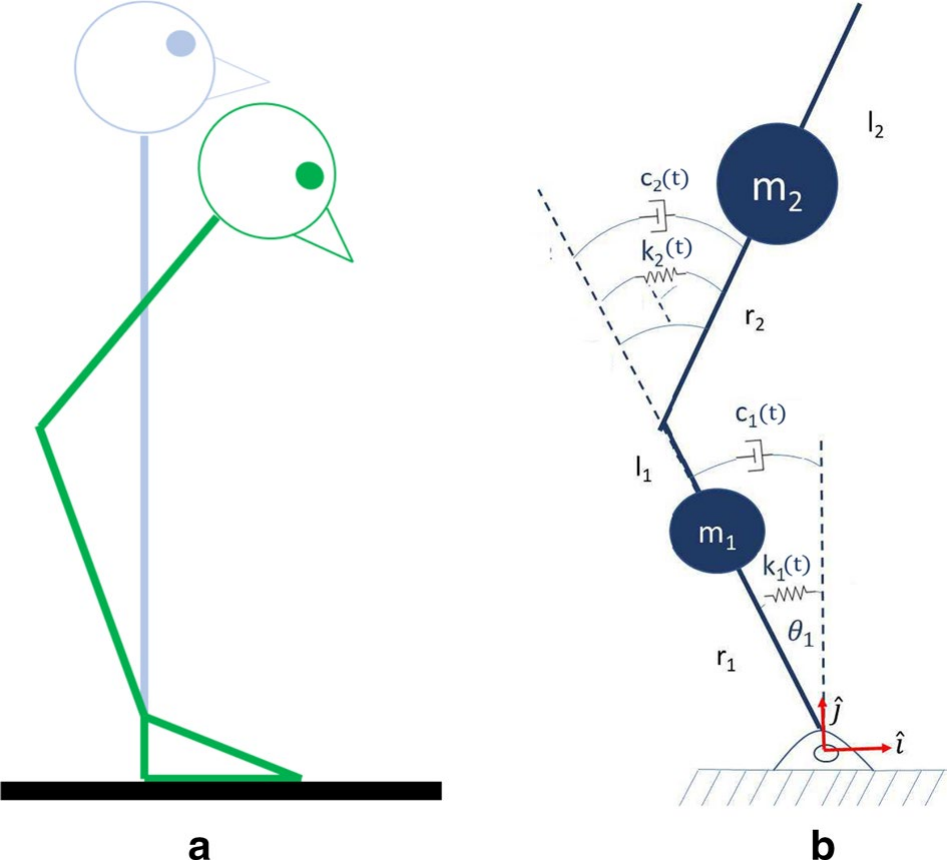
Mechanical representation of the hip balancing strategy:** a** A sagittal view of the hip balancing strategy, while** b** is a representation of the human body modeled as a double inverted pendulum

A balancing human on the sagittal plane has been modeled using a second order dynamic model. Joints were assumed as time-varying Kelvin–Voight models including a set of visco-elastic parameters whose values can be estimated by measuring joint positions. The model can be seen in Fig. 1. Both joint stiffness (*k*) and damping (*b*) were considered. Additionally, an estimation procedure of constant and time-varying visco-elastic parameters by means of the least squares method (LSM) and the Kalman filter (KF), respectively, were proposed and tested. Three distinct experiments were performed.

### Estimated values for the mechanical prototype

**Fig. 2:**
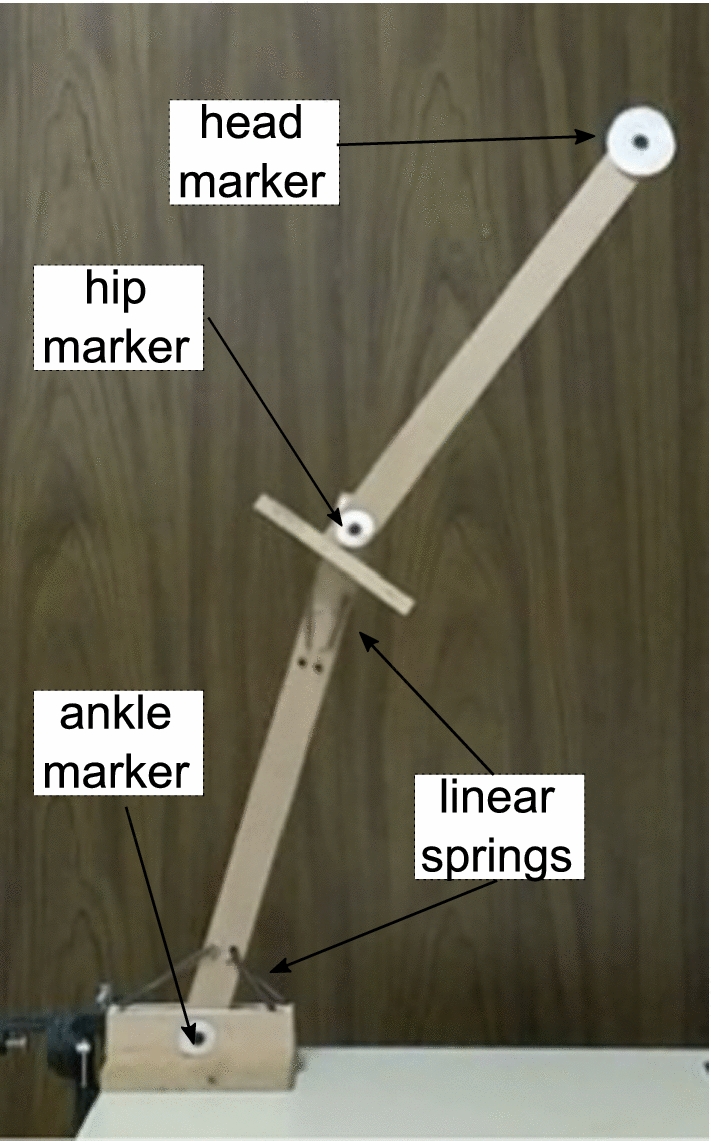
Prototype built to validate the parameter estimation procedure. The stiffness at the joints was simulated using linear springs between the joints. To reduce friction, all contact surfaces were covered with Teflon

In order to validate the estimation procedure, a mechanical prototype of known dynamics was built using linear springs and pure rotation joints (see Fig. 2). This resulted on a non-linear stiffness around the joints. The Kalman filter acts upon two main sources of noise, which are assumed uncorrelated. As the dynamic model of this mechanical prototype is well known, it allowed us to better tune the KF component acting on the measurement noise accounting for the inaccuracies of the instrumental setup.

**Fig. 3:**
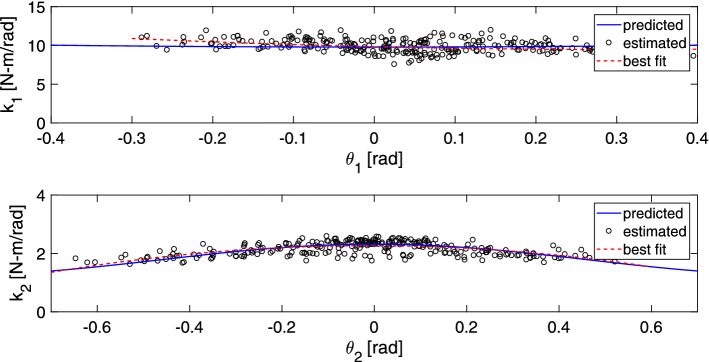
Stiffness around the joints axis of the constructed double pendulum. The blue line shows the estimated stiffness value considering constant linear springs and a changing moment arm. The black markers represent the estimated parameter obtained using a KF. The dashed red line shows the best fit of the estimated parameter values as a function of joint angle

A KF was used to obtain a non-constant estimate of each of the visco-elastic parameters ($k_1(t)$, $k_2(t)$, $b_1(t)$, $b_2(t)$). The estimated values for joint stiffness of the prototype are shown in Fig. 3 as black round markers. These estimated values closely resemble the expected stiffness values computed as a function of the springs’ moment arm shown with a blue line. The dashed red line gives the best fit of the estimated values as a function of joint angle. The estimated values obtained using the KF method are 9.4 Nm/rad and 1.9 Nm/rad for the ankle and hip joints respectively. Compared to 9.38 Nm/rad and 1.88 Nm/rad of the calibrated springs, these values represent the stiffness of the springs unaltered by the influence of the non-linearity due to non-constant moment arm. This shows the effectiveness of the proposed KF method for estimation of non-constant visco-elastic parameters.

### Estimated values for the simulated motion capture data

In order to determine the best procedure for estimating the human visco-elastic parameters a dynamic simulation of a balancing human (mass = 85 kg, height = 1.7 m) was created. The simulated human dynamic parameters were approximated from anthropometric data [30], while its joint stiffness was defined as a time changing value with magnitude based on existing literature [6].

After artificially adding noise to the measured joint angles, the visco-elastic parameters were estimated using both the LSM and KF approaches.

Using the LSM, the estimated values were:

1 $$\begin{aligned} \hat{\varvec{{\lambda }}}_{\text{LSM}} = {\left[ \begin{array}{cccc} 1191.1~\text{Nms/rad}&27.5~\text{Nms/rad}&547.4~\text{Nms/rad}&20.3~\text{Nms/rad}\end{array}\right] }^{\text{T}} \end{aligned}$$

These constant values are within the maximum and minimum expected values for the parameters and do not account for their time changing nature.

**Fig. 4:**
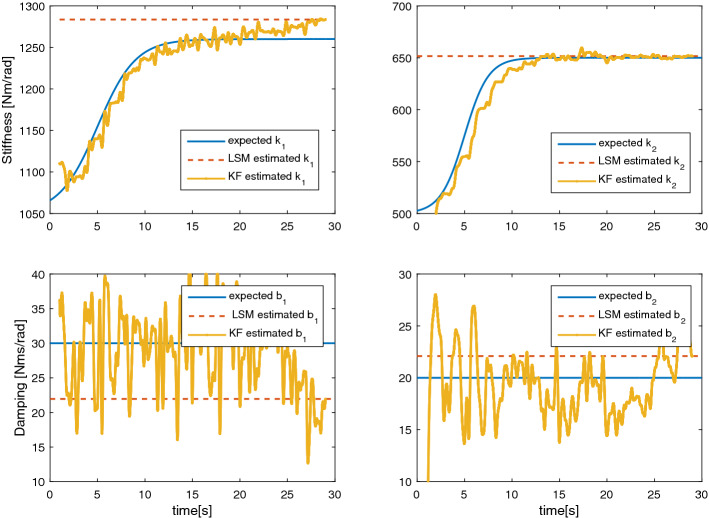
Estimated values for the visco-elastic parameters obtained using simulated data. The continuous blue line is the parameter value used to generate the data set, the dashed red line is parameter value estimated using a least squares approach, and the yellow line gives the time changing values obtained using a Kalman filter estimation

In fact, $\hat{\varvec{{\lambda }}}_{\text{LSM}}$ values are computed as an average over time of the time-varying visco-elastic parameters. The smaller the time-dependent changes are, the closer $\hat{\varvec{{\lambda }}}_{\text{LSM}}$ should be to the actual values. Figure 4 shows $\hat{\varvec{{\lambda }}}_{\text{LSM}}$ compared to the real parameter vector.

We propose a clear example in which the stiffness varies from a resting value to a constant value capable of maintaining the stability of the system. It can be seen that the regression method underestimates this critical value providing a constant value for the whole duration.

**Table 1:** Estimation error for the second-order model’s visco-elastic parameters

	$\hat{\varvec{{\lambda }}}_{\text{LSM}}$	$\hat{\varvec{{\lambda }}}_{\text{KF}}$	
$\mathtt {Mean}(\varvec{{\rho }})$	4.2E−3	− 1.4E−2	Nm
$\mathtt {RMSE}(\varvec{{\rho}})$	2.1E−1	2.5E−3	Nm

Table 1 gives the estimation error achieved when the estimated parameters are used to reconstruct the measured torque. For the sake of readability, we report the average and root mean squared error (RMSE) of the said error.

Using the KF approach, the evolution of the time-changing estimated vector ($\hat{\varvec{{\lambda }}}_{\text{KF}}$) is shown in Fig. 4. Additionally, Table 1 gives the estimation error obtained with this vector. Finally, Table 2 gives the RMSE error of the estimated time-varying parameters against the values used for generating the simulated data. In this way, it was possible to tune the KF yielding good results despite assuming incorrect process dynamics.

**Table 2:** Parameter estimation RMSE error for the simulated human

Parameter	$\mathtt {RMSE}$	$\mathtt {NRMSE}$ (%)
$k_{1}$	19.53 Nm/rad	1.59
$b_{1}$	9.26 Nms/rad	1.47
$k_{2}$	4.15 Nm/rad	13.86
$b_{2}$	2.36 Nms/rad	11.84

Table 2 shows the errors obtained using data from the simulated experiment:

We observe a fairly good tracking of joint stiffness parameters ($k_{1}$ and $k_{2}$) despite assuming the parameters to be constant when the KF was initialized. The estimation error for the damping coefficient ($b_{1}$ and $b_{2}$) is larger, possibly indicating a high sensitivity to the estimation of these parameters due to the covariance estimation while the stiffness values are changing.

**Fig. 5:**
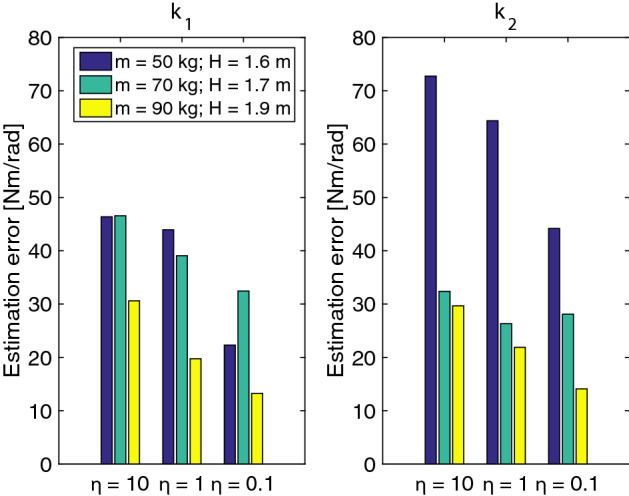
Estimation errors for three simulated subjects with three different rise velocities on the joint stiffness parameters

To explore the limits of the simulated model and the proposed KF, nine additional simulations were performed. These new simulations correspond to three new subjects (masses = 50, 70 and 90 kg; heights: 1.6, 1.7, 1.9 m, respectively). Additionally, three conditions were considered for the time-varying characteristics of the visco-elastic parameters ($\eta ={10,1,0.1}$) Fig. 5 shows the root mean squared error of the estimation (rmse) for the stiffness. With the proposed KF, the largest rmse corresponds to a 14% over the value of $k_2$ and only a 4% on $k_1$. We found that as the size of the subject increases, the estimated values are more accurate.

### Estimating the values of visco-elastic parameters of human subjects

The KF parameter estimation method was used on human subject data in order to determine the range of possible values for the visco-elastic parameters while balancing. A total of 10 subjects participated on the study.

**Fig. 6:**
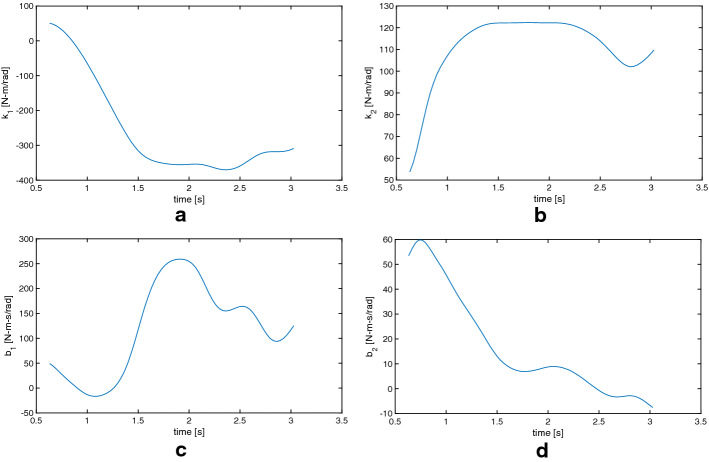
Experimental visco-elastic parameters estimation for a representative subject.** a** Stiffness of the ankle joint $k_{1}$.** b** Stiffness of the hip joint $k_{2}$.** c** Damping of the ankle joint $c_{1}$.** d** Damping of the hip joint $c_{2}$

Figure 6 shows the estimated parameters for one subject. It can be observed that the stiffness of the ankle joint $k_{1}$ takes negative values while all other parameters remain positive. This behavior could be explained as neuro-muscular-mediated activity during the motion.

**Fig. 7:**
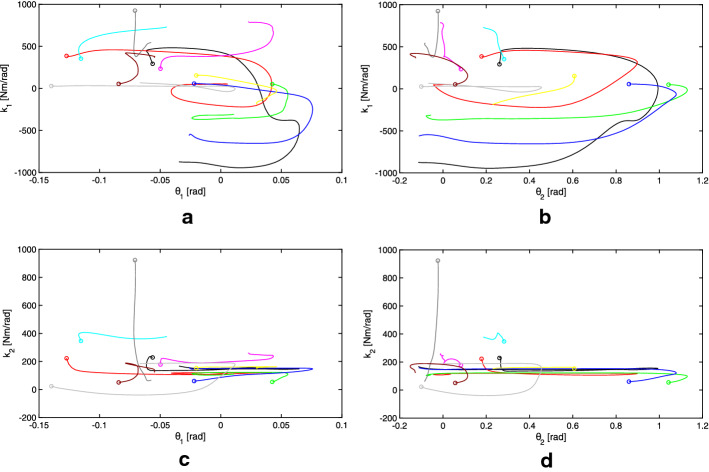
Estimated joint stiffness as a function of joint angle for 10 human subjects. Each subject is represented by a different colored line while the large circular marker represents the estimation for the parameter value at the first iterations of the Kalman filter.** a** Stiffness of the ankle joint $k_{1}$ with respect to the measured ankle joint angle.** b** Stiffness of the ankle joint $k_{1}$ with respect to the measured hip joint angle.** c** Stiffness of the hip joint $k_{2}$ with respect to the measured ankle joint angle.** d** Stiffness of the ankle joint $k_{2}$ with respect to the measured hip joint angle

Figure 7 shows the estimated value of the four visco-elastic parameters with respect to joint angles for all 10 subjects, each represented by a different colored line. The first estimated value, as the KF first begins to update using new measurements, is represented by a large circular marker. There appears to be a consistent behavior for most of the tested subjects. Note that the hip stiffness ($k_2$) remains relatively constant throughout the motion and does not seem to change between subjects. The ankle stiffness ($k_1$) decreases rapidly, reaching a negative value, as the hip joint angle reaches its maximum value.

Using Fig. 7, we observe that the subjects can be roughly grouped into two distinct categories. The first group maintains a constant joint stiffness and shows a small angular displacement, while the second group shows a larger displacement followed by activation of the joint musculature (observed as a negative stiffness). This could be a result of several factors, such as: age, subject mass, and perturbation energy. Future work should focus on explaining these differences in balancing strategies.

To test the control strategy involving muscle activation, we applied it to a simulated double pendulum. As detailed in [31], the simulated pendulum begins with a positive ankle stiffness which later changes into negative as the subject activates joint musculature. This change may allow for a faster stabilization as described by Morasso and Sanguineti [9]. The simulation performed is meant to test if this stiffness sign switching strategy can balance the subject.

As a first approximation the sign of the ankle stiffness ($k_1$) was chosen as a function of the leg’s angular position and velocity [31]. Conversely, the hip stiffness was keep positive and constant throughout the motion.

The simulated subject has a height of 1.7 m and a mass of 85 kg. The mass and dimensions of the model’s segments were obtained from literature anthropometric data for males [30]. The equations of motion were solved using MATLAB via a fourth-order Runge–Kutta method with a fixed time step of 10 ms. The simulation ran for a total of 3 s, simulation time. Initial conditions for the joint angles were consistent with the *hold and release* paradigm used during the experiments. In this case $\theta _1 = -0.06$ rads, $\theta _2 = 0.26$ rads, ${\dot{\theta }}_1 = {\dot{\theta }}_2=0$ rad/s.

**Fig. 8:**
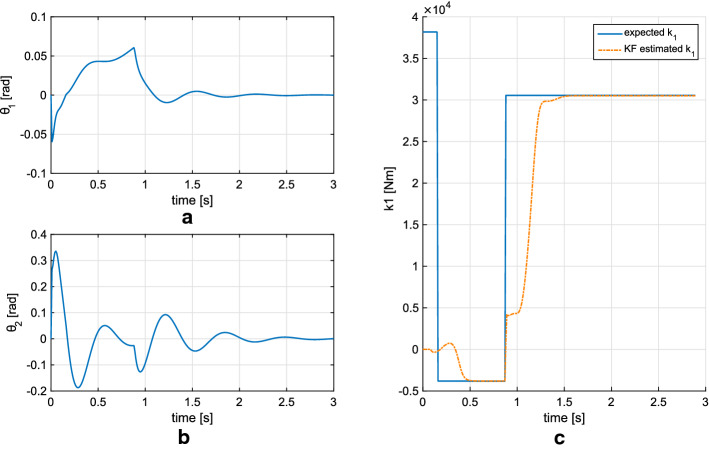
Estimated segment orientation obtained through a simulation. As shown in** c** the ankle stiffness was allowed to take negative values. Results** a** and** b** show that this strategy can result in a stable system. Additionally** c** shows that the implemented KF is capable of tracking joint stiffness very rapidly

Figure 8 shows the calculated joint displacement values for the simulated human, showing that stabilization in an upright posture is possible. Additionally, Fig. 8 shows the imposed joint stiffness as a function of time, and its estimated value when using the proposed KF. Note the filter’s fast response to step-like changes; while these are unlikely to occur in actual human experiments, they show the filter’s ability for tracking changes in stiffness (up to 10 kNm/s in this example).

## Discussion

In this paper, we analyzed a two degrees of freedom model of human standing actuated at each link. The torque applied at the joints is assumed to be a time-varying first-order model. That is, torque is generated proportionally to the time-varying angular displacement and angular velocity but the coefficients can change as a function of time. We have assumed that only the coefficient of proportionality to the angular displacement (i.e., stiffness) is time-varying. This allows us to reduce a much more complex system as a time-varying second-order system [32]. Note, that the equations utilized for our model are non-linear and have not been linearized due to the large variation of the angles in the act of recovering from a fall. This work focused mainly on the presentation of a vibrational model to represent the recovery from a fall in standing humans. It also provided a validation to a time-based system-identification technique using human data, simulations, and a physical prototype with joint stiffness that can change as function of time and angular displacement of either joint.

The changing moment arm for the linear springs in the prototype was modeled as a function of the segments’ orientation and used to calculate the “predicted stiffness” in Fig. 2. This provided a ground truth against which to compare the experimental results. Indeed, for the prototype the spring stiffness is constant but the joint stiffness changes as function of the non-linear moment arm. We are able to capture this in Fig. 2. For the human measurements, the model is affected not only by the non-linearity of the moment arms but the stiffness can also change as a function of time since muscle activation is a function of time. Further validation could be provided by using the KF to merge additional measurements of the muscle activation and assuming a correlation between these two variables. As the dynamics of the double pendulum are well understood, it provided an ideal platform for testing the KF and estimating the measurement noise of our motion capture system. This value would later be used to the experimental data obtained from the human subject.

An initial set of simulations allowed us to ascertain the accuracy of the assumed parameter dynamics. The simulated data were created using Body Segment Parameters (BSPs) close to the average of our subject population. The KF process noise was estimated here and this value would later be used to the experimental data obtained from the human subject. This simulation confirmed the stability of the filter and the ability to track a continuous time-varying stiffness, as shown in Fig. 4. An additional simulation, seen in Fig. 8, verified that a sequence of positive and negative stiffness could provide a physical solution to maintain balance. Furthermore, by using a discontinuous stiffness signal, we observed that the maximum stiffness rate of change trackable by the KF far exceeds any physiological rate of change for the average BSPs. Additionally, we performed a set of simulated experiments where the time rise of the stiffness and inertial properties of the system have been changed to assess the sensitivity of the filter (Fig. 5). While maintaining a body mass index (BMI) ranging from 19.5 to 24.5 kg/m^2^, we observe that systems with increased inertia provide more accurate tracking results of the stiffness. Indeed, an increase in inertia reduces the natural frequencies of the system, making the evolution of the state variables slower.

In our experimental results on human subjects, we observed a well-defined synchronization of stiffness modulation between the stiffness of one joint (in this case, the ankle) and the displacement of the other joint (hip angle). It is noteworthy to highlight that we have observed both positive and negative stiffness. A positive stiffness creates a torque that opposes the displacement of the link and tends to bring the link back to the vertical where the force of gravity has no moment arm. A negative stiffness, on the other hand, has a destabilizing effect on the joints and it is acting upon moving the link away from the vertical (i.e., the equilibrium position). When modeling, no assumption was made regarding the sign of the joint stiffness. Indeed, negative stiffness can be obtained even if the muscles can only apply force in one direction. Assuming that each muscle has a basal non-zero activation each muscle is in a pre-tensioned state. If a muscle is stretched a standard segmental reflex induces an increase in muscle activation. This increased muscle activation is generated by the stretching of the muscle fibers. Consequently, the muscle will contract applying a force opposite to the direction of the stretch. In the convention utilized in the paper this is represented as a positive stiffness that tends to re-establish the initial equilibrium position. On the other hand, the central nervous system is also capable of inhibitory effects usually via the Golgi tendon organs (GTOs). These shortening reflexes inhibit the muscle activation when the muscle is stretched. Thus, according to our convention this will generate a negative stiffness since the force decreases as the muscle is stretched, bringing the system temporarily away from the initial equilibrium condition. Since the joint stiffness needs to be generated by opposing muscles (because a muscle can only pull) we have muscle couples acting in synchrony controlled by heteronomous reflexes. For positive stiffness we will have that the muscle that is stretched increases the force and the opposing muscle will shorten and decrease its force. For negative stiffness we will have that the shortening muscle will increase its force while the stretched side will decrease the force.

Interestingly, the destabilizing effect introduced by the negative stiffness at the ankle tends to stabilize the system as a whole. Indeed, we see that the stiffness at the ankle decreases (or becomes negative) when the angle at the hip reaches its maximum (see Fig. 7). This creates a “whip” effect on the structure. By inverting the direction of the torque at the ankle when the hip angle is maximum, the system takes advantage of the interaction torque created by the inertial properties of the system. A sudden change in ankle’s angle pushing it away from the equilibrium induces a negative interaction torque around the hip due to the terms outside the diagonal in (11) thus moving the center of gravity of the trunk link closer to vertical and more stable position. This is in line with the type of “throw-and-catch” control which has been previously observed [9, 10] where it was posited that a torque burst at the ankle would occur at extreme angles in the direction opposite to the movements. This would allow the ankle angular velocity to change sign. It was also observed that, during the oscillation phase, the ankle’s stiffness would be very low, allowing for the angle to change from one side to the other of the vertical. Our finding partially supports this view, but the addition of the second link in the double-pendulum model highlights that the torque burst at the ankle actually has the same direction of the angular velocity but induces a change in angular velocity at the hip and thus the stabilization of a two degrees of freedom system by simply controlling one degree of freedom.

Regarding the stiffness at the hip, an interesting observation can be obtained from the joint’s anatomy. It is known that the hip flexors on the back are quite large. For example the gluteal group (*gluteus medius* and *gluteus maximus*) wrap around the dorsal side of hip joint and can provide a very large force and, hence, a large stiffness. On the ventral side of the human hip the psoas is the primary hip flexor, assisted by the iliacus. The pectineus, the adductors longus, brevis, and magnus, as well as the tensor *fasciae latae* are also involved in flexion. These primary flexors are small when compared to the extensors. Since the hip stiffness around an axis orthogonal to the sagittal plane is regulated by the co-contraction of antagonistic flexors and extensors, the hip stiffness needs to be regulated in order to not exceed the force that the smaller muscles are capable of applying. This is also correlated to the specific geometrical structure of the hip which allows a large displacement while bending forward from the erected position, but little joint displacement bending backward due to limitation in the articulation. Bending forward is regulated by the large gluteal muscle group but the impossibility to bend backward makes large flexor unnecessary. Therefore, it is physiologically plausible that during a recovery from a fall subjects would not increase the stiffness at the hip but would simply try to use the stronger muscle (i.e., the extensor muscles on the back) to recover balance. Therefore, it makes sense to have instability induced by the sudden rotation off the ankle to induce the upper part of the body to lean forward, hence using the larger muscles for stabilizing the system.

Direct measurements of the ankle stiffness [10, 16] have pointed out the insufficiency of using the intrinsic muscle stiffness as the sole mechanism to balance quiet standing. Many researches have proposed the use of classical lineal continuous-time feedback control to accomplish this task [18, 33–37]. However, since the motor cortex mediates the voluntary control of movement, the somatosensory signal coming from the different joints needs to be transmitted to the cortex, and then, after processing, a motor command is to be sent to the muscles controlling each joint. This would imply that the controller is working with significant instability-inducing delays in the feedback loops that are different for each joint and are the longest for the actuation of the ankle. These delays are difficult to reconcile with the stable behavior observed and would require a high cognitive load to be managed appropriately. A recent simulation-based study [38] has proposed a control mechanism where the ankle is controlled via an intermittent control, while the hip is control using an intrinsic stiffness modality. It was hypothesized that the critical stiffness value to maintain the stability of the trunk hinged at the hip joint is much smaller than for the whole body hinged at the ankle. This is due to the lower mass of the upper part of the body and the shorter distance of its center of mass from the hip’s center of rotation. The physiological plausibility of this assumption is supported by the lack of correlation between trunk movements and the activity of the muscles around the hips [39]. Our study is the first to experimentally support this hypothesis and see direct evidence that a double-pendulum model may be stabilized by changing only the ankle’s stiffness.

## Conclusions

We presented a second-order dynamic model suitable for the estimation of the ankle and hip neuro-mechanical visco-elastic parameters. This model was evaluated using a simplified mechanical prototype and simulation data with good overall results. This allowed us to reach a good level of confidence that the model can be used to estimate the visco-elastic parameters of human subjects with accuracy. Finally, the estimation procedure was applied to human motion capture data. There appears to be a consistent strategy, implemented by nine out of the 10 subjects, for balancing using the hip strategy. In particular, the hip stiffness remains constant while the angle stiffness is modulated in order to regain balance. Furthermore, we have tested the observed strategy on a simulation study and have found it capable of stabilizing the double-pendulum human model.

The estimation procedure we detail can be easily implemented in a clinical setting and could be used to determine changed in the balancing strategy of an individual. This could in turn be used as a diagnosis tool to determine balance degradation or improvement due to aging, injury, or neuro-degenerative diseases.

## Methods

### Second order model for a balancing human

An inverted-pendulum model such as the one shown in Fig. 1a can be used to represent the movement of the hip and ankle during quiet standing. The two segments representing the trunk and the legs are actuated by muscles–tendon units with different visco-elastic neuro-mechanical properties [40]. Such properties can be estimated by observing the model’s dynamics over time. Based on Fig. 1b, the equations that govern the system’s motion can be obtained trough dynamic modeling. The following deduction, made through the Euler–Lagrange methodology [41], defines $m_{i}$ as the mass of segment *i*, $l_{i}$ as the distance measured between two consecutive joints, $r_{i}$ as the distance from the distal joint to the segment’s center of mass position, and $k_{i}$ and $b_{i}$ are the joint stiffness and damping, respectively. Note that the stiffness and damping of the joints are produced by the muscle around said joints. Because there are no bi-articular muscles spanning from below the ankle to above the hip, we are not including any cross-joint stiffness or damping.

The position of each mass in the system ($\varvec{{\mathrm{r}}}_{m_{i}}$) is determined as a function of the generalized coordinates $$\varvec{{\mathrm{q}}}=\begin{bmatrix}\theta _{1}&\theta _{2}\end{bmatrix}^{\text{T}}$$ as:

2 $$\begin{aligned} \varvec{{\mathrm{r}}}_{m_1}= & {} -r_{1}\sin {\theta _{1}} \varvec{{\hat{i}}} + r_{1} \cos \theta _{1}\varvec{{\hat{j}}} \end{aligned}$$

3 $$\begin{aligned} \varvec{{\mathrm{r}}}_{m_2} &= (-l_{1} \sin \theta _{1} + r_{2} \sin \theta _{21}) \varvec{{\hat{i}}}\\& \quad +(l_{1}\cos \theta _{1} + r_{2}\cos \theta _{21}) \varvec{{\hat{j}}} \end{aligned}$$

where $\theta _{21} = \theta _2 - \theta _1$ and $[\varvec{{\hat{i}}}$, $\varvec{{\hat{j}}}]$ are the cosine directors shown in Fig. 1b.

The system’s total kinetic energy (*T*) is obtained using the velocity of each mass calculated as the first time derivative of (2) and (3). The total kinetic energy of the system is then:

4 $$\begin{aligned} T= & {} \frac{1}{2} \left( m_{1}r_{1}^{2}{\dot{\theta }}_{1}^{2} + I_{1}{\dot{\theta }}_{2}^{2} + I_{2} {\dot{\theta }}_{21}^{2}\right. \nonumber \\&+\left. m_{2}\left( l_{1}^{2}{\dot{\theta }}_{1}^{2} + r_{2}^{2} {\dot{\theta }}_{21} ^{2} - 2l_{1}l_{2}\dot{\theta _{1}} {\dot{\theta }}_{21} \cos \theta _{2}\right) \right) \end{aligned}$$

where $I_{i}$ is the segment’s moment of inertia with respect to the axis of rotation of joint *i*.

The system’s potential energy (*V*), including that stored by the elastic elements is found to be:

5 $$\begin{aligned} V= & {} m_{1}gr_{1}\cos \theta _{1} + m_{2}g\left( l_{1}\cos \theta _{1}+r_{2}\cos \theta _{21}\right) \nonumber \\&+\frac{1}{2} \left( k_{1}\theta _1^2+k_2\theta _{2}^{2}\right) \end{aligned}$$

where $k_{i}$ is the time-dependent stiffness of the ankle and hip joints and *g* represents the acceleration of gravity.

Finally, it is necessary to consider the energy dissipated by the dampers [42] as a function of the time-varying damping coefficients $b_i$. The dissipated energy is given by:

6 $$\begin{aligned} D = \frac{1}{2} b_{1} {\dot{\theta }}_{1}^{2} +\frac{1}{2} b_{2} {\dot{\theta }}_{2}^{2}. \end{aligned}$$

The Lagrangian is then defined as:

7 $$\begin{aligned} L=T-V. \end{aligned}$$

The motion of the system, taking its dynamic properties into consideration [42], can be obtained as:

8 $$\begin{aligned} \frac{d}{{\text{d}}t} \frac{\partial L}{\partial \dot{\hat{q_{k}}}} - \frac{\partial L}{\partial \hat{q_{k}}} + \frac{\partial D}{\partial \dot{\hat{q_{k}}}} = {\hat{Q}}_{k}, \end{aligned}$$

where

9 $$\begin{aligned} \hat{\varvec{{\mathrm{Q}}}}_{k} = \sum _{j=1}^{p} F_{j} \cdot \frac{ \partial r_{j} }{ \partial {\hat{q}}_k}, \end{aligned}$$

with $F_{j}$ representing the external forces applied to the system.

Under no external forces (${\hat{Q}}_{k}=0$), by substituting into (7) and (6) in (8), the double inverted pendulum can be described by the second-order non-linear system:

10 $$\begin{aligned} {\mathbf {M}}(q)\ddot{q}+{\mathbf {C}}(q,{\dot{q}}){\dot{q}}+{\mathbf {G}}(q)=\tau (q), \end{aligned}$$

where

11 $$\begin{aligned} \varvec{{\mathrm{q}}}= & {} {\left[ \begin{array}{cc} \theta _1&\theta _2\end{array}\right] }^{\text{T}} \nonumber \\ \varvec{\mathrm {M}}= & {} \left[ \begin{array}{cc} \alpha +2\beta \cos \theta _{2} &{} -\left( \gamma +\beta \cos \theta _{2}\right) \\ -\left( \gamma +\beta \cos \theta _{2}\right) &{} \gamma \end{array}\right], \end{aligned}$$

12 $$\begin{aligned} \varvec{\mathrm {C}}= & {} \left[ \begin{array}{cc} - 2 \beta {\dot{\theta }}_{2} \sin \theta _{2} &{} \beta {\dot{\theta }}_{2} \sin \theta _{2} \\ \beta {\dot{\theta }}_{1} \sin \theta _{2} &{} 0 \end{array}\right] \nonumber \\ \varvec{\mathrm {G}}= & {} \left[ \begin{array}{c} \epsilon g \sin \theta _{21} - \delta g \sin \theta _{1}\\ -\epsilon g \sin \theta _{21} \end{array}\right] \nonumber \\ \varvec{{\tau }}= & {} -\left[ \begin{array}{c} k_1 \theta _1 + b_1 {\dot{\theta }}_1 \\ k_2 \theta _2 + b_2 {\dot{\theta }}_2 \end{array}\right], \end{aligned}$$

13 $$\begin{aligned} \alpha= & {} I_{1}+I_{2}+m_{1}r_{1}^{2}+m_{2}\left( l_{1}^{2}+r_{2}^{2}\right) \nonumber \\ \beta= & {} m_{2}l_{1}r_{2} \nonumber \\ \gamma= & {} I_{2}+m_{2}r_{2}^{2} \nonumber \\ \delta= & {} m_{1} r_{1} + m_{2} l_{1}\nonumber \\ \epsilon= & {} m_{2}r_{2}, \end{aligned}$$

### Parameter estimation

To estimate the value of the neuro-mechanical visco-elastic parameters, it is convenient to rewrite the second-order model (10) as a linear function of the visco-elastic parameters:

14 $$\begin{aligned} \varvec{{\mathrm{Z}}}= & {} \varvec{\mathrm {H}}\varvec{{\lambda }}, \end{aligned}$$

15 $$\begin{aligned} \varvec{\mathrm {H}}= & {} \left[ \begin{array}{cccc} \theta _{1} &{} {\dot{\theta }}_1 &{} 0 &{} 0\\ 0 &{} 0 &{} \theta _2 &{} {\dot{\theta }}_2 \end{array}\right], \end{aligned}$$

16 $$\begin{aligned} \varvec{{\mathrm{Z}}}= & {} -\varvec{{\tau }}, \end{aligned}$$

where $\varvec{{\lambda }}$ contains the model’s, time-varying, neuro-mechanical stiffness and damping parameters:

17 $$\begin{aligned} \varvec{{\lambda }}(t) = {\left[ \begin{array}{cccc} k_1(t)&b_1(t)&k_2(t)&b_2(t)\end{array}\right]}.^{\text{T}} \end{aligned}$$

$\varvec{{\mathrm{Z}}}$ is a vector of torques computed through the model’s dynamics, $\varvec{\mathrm {H}}$ is known as the configuration matrix and relates the model’s parameters to the torque vector.

For the linear system given in (14), an estimated parameter vector, $\hat{\varvec{{\lambda }}}$ is proposed such that:

18 $$\begin{aligned} \varvec{{\mathrm{Z}}} = \varvec{\mathrm {H}}\hat{\varvec{{\lambda }}}(t) + \varvec{{\rho }}, \end{aligned}$$

and $\varvec{{\rho }}$ represents the estimation error.

*Least squares estimation* When $\varvec{{\lambda }}$ is considered to be a constant value, it is possible to find a solution to (18) using any standard linear regression techniques.

An estimated parameter vector, $\hat{\varvec{{\lambda }}}$, can be obtained using the ordinary least squares approach. This approach has been shown to minimize the Euclidean norm of the estimation error, $\varvec{{\rho }}$ [43]. A simple computation can be performed by making use of the Moore–Penrose pseudoinverse [44]:

19 $$\begin{aligned} \varvec{\mathrm {H}}^+= & {} {\left( {\varvec{\mathrm {H}}}^{\text{T}}\varvec{\mathrm {H}}\right) }^{-1}{\varvec{\mathrm {H}}},^{\text{T}} \end{aligned}$$

20 $$\begin{aligned} \hat{\varvec{{\lambda }}}= & {} \varvec{\mathrm {H}}^{+}\varvec{{\mathrm{Z}}}. \end{aligned}$$

Ideally, there should be *at least* enough linearly independent measurements such that $\varvec{\mathrm {H}}$ is invertible. In practice, enough measurements can be joined to create an overdetermined system [45].

*Kalman filter* When $\varvec{{\lambda }}(t)$ is considered to be non-constant, a recursive approach is better suited. One commonly used method is the Kalman filter (KF), as it can correct the current parameter estimation based on new measurements [46]. The KF equations are written as follows:

21 $$\begin{aligned} \hat{\varvec{{\lambda }}}_{k}^{+}= & {} \varvec{\mathrm {A}} {\hat{\lambda }}_{k}^{-} + \varvec{\mathrm {B}} \mu _{k}, \end{aligned}$$

22 $$\begin{aligned} \varvec{\mathrm {P}}_{k}^{+}= & {} \varvec{\mathrm {A}} \varvec{\mathrm {P}}_{k}^{-}{\varvec{\mathrm {A}}}^{\text{T}}+\varvec{\mathrm {Q}}, \end{aligned}$$

23 $$\begin{aligned} \varvec{\mathrm {K}}_{k}= & {} \varvec{\mathrm {P}}_{k}^{-} {\varvec{\mathrm {H}}}{\text{T}} {\left( \varvec{\mathrm {H}}\varvec{\mathrm {P}}_{k}^{-}{\varvec{\mathrm {H}}}{\text{T}}+\varvec{\mathrm {R}}\right) }^{-1}, \end{aligned}$$

24 $$\begin{aligned} \hat{\varvec{{\lambda }}}_{k}= & {} \hat{\varvec{{\lambda }}}_{k}^{-}+\varvec{\mathrm {K}}_{k}\left( \varvec{{\mathrm{Z}}}_{k} - {\mathbf {H}}\hat{\varvec{{\lambda }}}_{k}^{-}\right), \end{aligned}$$

25 $$\begin{aligned} \varvec{\mathrm {P}}_{k}= & {} \left( \varvec{\mathrm {I}}-\varvec{\mathrm {K}}_{k}\varvec{\mathrm {H}}\right) {\varvec{\mathrm {P}}_{k}^{-}}, \end{aligned}$$

where $\varvec{\mathrm {A}}$ and $\varvec{\mathrm {B}}$ determine how the model’s parameters ($\varvec{{\lambda }}_k$) change over the discretized time; $\varvec{\mathrm {P}}_k$ is the estimation covariance, $\varvec{\mathrm {Q}}$ is the model covariance, while $\varvec{\mathrm {R}}$ is the measurement covariance. While the KF estimation may fail when $\varvec{\mathrm {A}}$, $\varvec{\mathrm {Q}}$, $\varvec{\mathrm {H}}$ and $\varvec{\mathrm {R}}$ are not exactly known, a commonly used strategy to increase the filter’s performance is to tune $\varvec{\mathrm {Q}}$ [46]. For the work presented here, $\varvec{\mathrm {Q}}$ has been chosen such that the dynamic parameters of a known simulation model can be suitably identified while assuming a known measurement error. Using the simulation data, it is possible to determine if the performance of the KF was acceptable. For the work presented here, we have assumed $\varvec{\mathrm {R}}_k$ as the one determined using the mechanical prototype, and $\varvec{\mathrm {A}}$ as an identity matrix. This last assumption means that the stiffness parameters should be constant. The process covariance matrix $\varvec{\mathrm {Q}}$ was tuned to reduce the Filter’s estimation error as measured by the $\mathtt {RMSE}$ (Table 2). This way, we hope that the measurement and model uncertainty values ($\varvec{\mathrm {R}}$ and $\varvec{\mathrm {Q}},$ respectively) used on the human subject data are a good approximation and the filter yields a correct estimate.

The estimation covariance ($\varvec{\mathrm {P}}_k$) can be used to determine current confidence in the estimated parameters. As mentioned previously, $\varvec{\mathrm {H}}$ is the related configuration matrix, $\varvec{{\mathrm{Z}}}_k$ is the measurement vector, as defined in (16) and (15), respectively. $\varvec{\mathrm {K}}_k$ is known as the Kalman gain and weighs the impact of incoming measurements in the estimated parameter values. Finally, $\varvec{\mathrm {I}}$ is an identity matrix of suitable size.

### Experimental validation of the proposed dynamic model

Three different experiments were performed to validate the proposed double-pendulum model. First, a mechanical prototype with non-constant joint stiffness was built and studied. Then, a simulation consistent with a human subject’s motion was performed. The neuro-mechanical visco-elastic parameters of the simulation were estimated assuming both constant and non-constant values and compared to their known values. Finally, using human motion data, the joint stiffness and damping parameters of 10 able-bodied subjects were estimated and analyzed. The following sections include details on each of these experiments.

**Table 3:** Measured model parameters for a double inverted pendulum

Parameter	Value		Parameter	Value	
$m_1$	0.25 kg		$k_1$	Approx. 9.38	Nm/rad
$m_2$	0.20 kg		$b_1$	Negligible	Nms/rad
$l_1$	0.53 m		$k_2$	Approx. 1.88	Nm/rad
$r_1$	0.27 m		$b_2$	Negligible	Nms/rad
$r_2$	0.23 m				

#### Validation of the proposed model with a mechanical prototype

A double inverted pendulum was built (see Fig. 2) using linear extension springs to stiffen the prototype’s joints. To reduce friction all contact surfaces were covered with Teflon tape. Other than joint contact friction and air resistance, no damping elements were considered. The length and mass of the prototype’s segments were measured and they are presented in Table 3.

Four linear springs with a constant stiffness of 850 N/m were used on the model’s first joint (which simulates the ankle). While at the neutral position ($\theta _1=\theta _2=0$), the springs have a moment arm of 74.2 mm around the joint. Similarly, two springs with a constant of 690 N/m were used on the second joint (representing the hip) having a moment arm of 37.1 mm around the joint while at the neutral position. It should be noted that the resting lengths of the springs are such that while the prototype is in motion the springs on one side of the ankle joint may become relaxed. The springs on both sides of the hip joint remain taut throughout. For this prototype, and for small angular displacements, it is expected that the rotational stiffness of each joint will be $k_r = n \cdot k_l r^2$, where $k_r$ is the rotational stiffness $k_l$ is the linear stiffness of the spring, *r* the corresponding moment arm and *n* is the total number of active springs. That is, assuming a constant linear behavior of the springs and small angular displacements, the approximated stiffness of the joints in the equilibrium position are expected to be of 9.38 Nm/rad and 1.88 Nm/rad for the ankle and hip joints, respectively.

However, as the pendulum’s segments move past each other, the length of moment arm to the joint changes as a function of the joint angle ($\theta _1$ and $\theta _2$). The changing moment arms were calculated based on the prototype’s geometry and were used to estimate the, non-constant, joint stiffness. Figure 3 shows, using a solid blue line, the expected stiffness around each joint as a function of the respective joint angle.

To estimate stiffness values for each of the prototype’s joints, its motion was recorded. During the experiment the pendulum was moved to a random initial position, held there at rest (${\dot{\theta }}_1 = {\dot{\theta }}_2 = 0$), and then suddenly released. The motion was recorded using CCD-cameras with a frame rate of 22.5 fps.

The orientation of each segment was measured by tracking the position of three passive markers (corresponding to the ankle, hip, and head) using the open source software KINOVEA [47]. A total of eight different trials with unique initial conditions were performed.

The data obtained were used to estimate the prototype’s joints stiffness using a KF. This allowed for the estimation of an angle-dependent stiffness. Since angular position is dependent upon time, it follows that joint stiffness is also time-varying.

The KF parameters $\varvec{\mathrm {A}}$, $\varvec{\mathrm {B}}$, and $\varvec{\mathrm {H}}$ are well known for this mechanical prototype. Since the behavior for the angle-dependent joint stiffness is also well understood, it is possible to use this knowledge to trust the model and find suitable values for $\varvec{\mathrm {R}}$ which characterize the measurement noise. This was done by running the KF with different candidate values for $\varvec{\mathrm {R}}$ until the KF performed as expected. The measurement covariance matrix obtained in this way should be suitable for use on the measurement setup used here.

#### Simulating human motion capture data

Before gathering human subject data, the parameter estimation procedure was tested in simulation. This way the actual parameter values would be known.

**Fig. 9:**
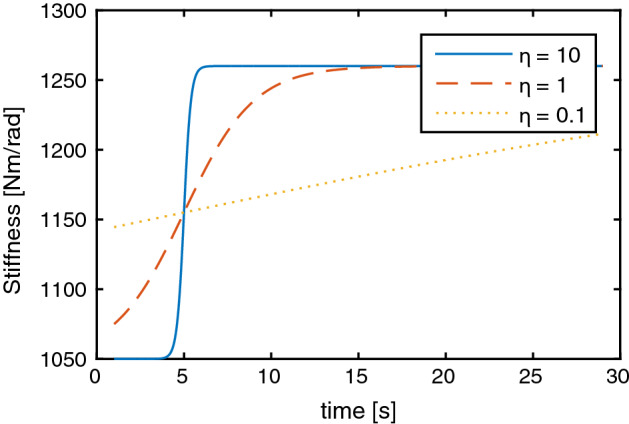
Different rise velocities of joint stiffness as a function of the parameter $\eta$

A numerical solution for the dynamic system presented in (10) was found using a fourth order Runge–Kutta approximation with a fixed step of 10 ms. The evolution of the neuro-mechanical dynamic system was simulated for 30 s. The simulation assumed a male subject of 85 kg and 1.7 m tall. The required geometric parameters ($m_{1}$, $m_{2}$, $l_{1}$, $r_{1}$, $l_{2}$), were obtained from anthropometric table data [30]. Time-varying values were proposed for the stiffness ($k_{1}(t)$, $k_{2}(t)$) using sigmoidal functions, while joint damping ($b_{1}(t)$, $b_{2}(t)$) were considered constant. Sigmoid functions were chosen to avoid discontinuities in the stiffness values, while still allowing for time-varying parameters. Their magnitude, as is that of the damping coefficients, are consistent to those reported by Coronado et al. [29]. The parameters and time-varying parameters used are detailed in Table 4. The effect of parameter $\eta$ on the rise of the visco-elastic parameters can be seen in Fig. 9.

**Table 4:** Simulation data for the estimation of the visco-elastic parameters

Parameter	Value	
*m* (total mass)	{50, 70, 85, 90}	kg
*H* (total height)	1.6, 1.7, 1.7, 1.8	m
$m_{1}$	0.322*m*	kg
$m_{2}$	0.678*m*	kg
$l_{1}$	0.53*H*	m
$r_{1}$	0.29*H*	m
$l_{2}$	0.18*H*	m
$\eta$	{10, 1, 1, 0.1}	
$k_{1}(t)$	$1050\left( 1+0.2\frac{1}{1+e^{-0.5 \eta (t-5)}}\right)$	Nm/rad
$b_{1}$	30	Nms/rad
$k_{2}(t)$	$500\left( 1+0.3\frac{1}{1+e^{-0.8 \eta (t-5)}}\right)$	Nm/rad
$b_{2}$	20	Nms/rad

Initial conditions shown in Table 5 were chosen to ensure that the ground projection of the model’s center of mass remained inside the area limited by the model’s feet.

**Table 5:** Initial conditions used for generating simulated data

Initial conditions		
$\theta _{1}$	− 0.10 rad	
${\dot{\theta }}_{1}$	0 rad/s	
$\theta _{2}$	0.22 rad	
${\dot{\theta }}_{2}$	0 rad/s	

**Fig. 10:**
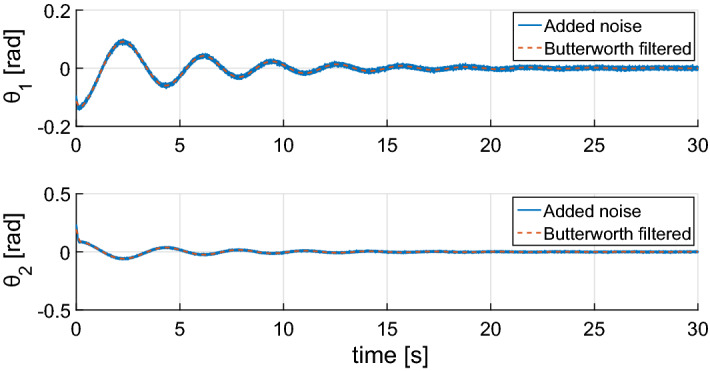
Simulated angular value for the orientation of each of the model’s segment. The full blue line represents the noisy angular values while the red dashed line shows the values after the application of the zero-phase Butterworth filter

With the intent to replicate motion capture data, the simulated angular displacements were corrupted with a large Gaussian noise ($\text{SNR} \approx -0.1\, \text{db}$). They represent the orientation of the body’s segments and were used to determine the time-varying system detailed in (18). A zero-phase, low-pass Butterworth filter with a cut-off frequency of 30 Hz was applied to the measured angular position in order to remove high-frequency noise [45]. Angular velocity and acceleration were then obtained in a numerically manner using the central difference approximation to avoid introducing phase shift. Both the noisy and filtered signals are shown in Fig. 10.

To evaluate the accuracy of the parameter estimation we define the error as $\varvec{{\rho }}_i = \varvec{{\mathrm{Z}}} - \varvec{\mathrm {H}}_i\hat{\varvec{{\lambda }}}_i$. This error is null when $\hat{\varvec{{\lambda }}}=\varvec{{\lambda }}$. Furthermore, as the time-changing values of the neuro-mechanical visco-elastic parameters are known, the root mean square error (RMSE) and normalized root mean square error (NRMSE) [48] were calculated.

To explore the limits of the simulation model and the proposed KF, nine more simulations were performed. They correspond to three new subjects (masses = 50, 70 and 90 kg; heights: 1.6, 1.7, 1.9 m respectively). Each subject was evaluated using three conditions the time and speed of change in the visco-elastic parameters (labeled as: fast, regular and slow). These conditions were applied directly to the sigmoidal function which determines the time characteristic of the parameter. The masses and height of the three simulated individuals were chosen to offer a range of equivalent inertia around the ankle joint. This way, a large range of oscillations are obtained before the eventual stabilization of the model.

### Human subject measurements

Motion capture data were obtained for 10 human subjects (three females and seven males, weight = 80.7 ± 11.7 kg and height = 1.67 ± 0.08 m). All subjects expressed their consent and participated freely in this study.

**Fig. 11:**
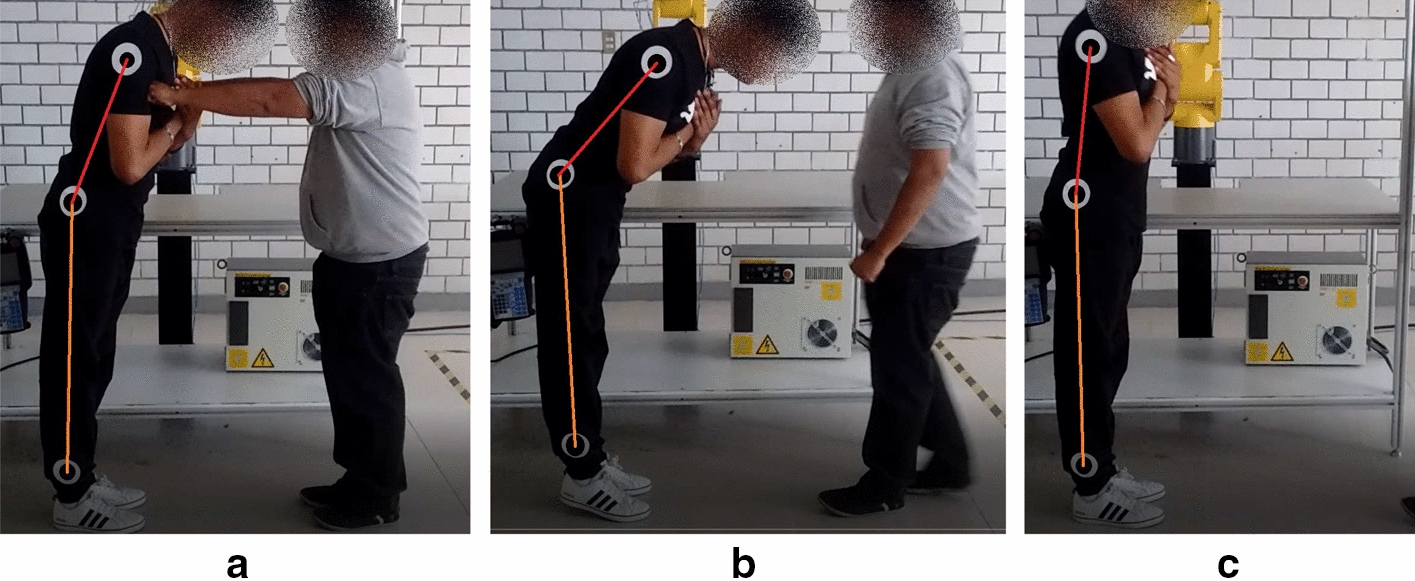
Experimental setup for the estimation of joint visco-elastic parameters using the *hold and release* method.** a** Shows the subject leaning onto the examiner.** b** The subject is suddenly released and must maintain balance in order to achieve a standing posture.** c** The subject has recovered a vertical posture

The experiment followed the *hold and release* paradigm [11], which consists of holding the subject at a certain hip and ankle angle, and suddenly releasing her, forcing her to initiate a recovery from a potential fall. The experiment procedure is shown in Fig. 11.

The angles between the body segments were calculated by detecting the position of markers placed on the ankle and hip joints, as well as one placed on the subject’s torso. Marker positions were measured using KINOVEA, sampled at an average frame rate of 20 Hz. KINOVEA has been found to be a “valid and reliable tool to measure distances [and angles] up to 5 m away” [49] in a study that focuses on performing measurements on a plane like the ones presented here. Additionally, Nord Adnan et al. [50] have compared KINOVEA to a HAWK-CORTEX system with similar conclusions regarding the reliability of the system for measuring motion on the sagittal plane.

As described previously, the angular measurements were low-pass filtered prior to use and time differentiated using central differences to avoid a phase shift in the data [45].

Finally, the neuro-mechanical visco-elastic parameters were estimated for each subject using the previously described LSM and KF approaches.

While the least squares approach using the pseudoinverse of the configuration matrix is convenient, it does not capture the time-varying characteristic imposed on the parameters. For this reason a KF-based estimation was also implemented. To implement the KF, it is necessary to propose values for $\varvec{\mathrm {A}}$, $\varvec{\mathrm {Q}}$, and $\varvec{\mathrm {R}}$. Having perfect knowledge of them, while recommended, is not always necessary. For the experiments presented here, $\varvec{\mathrm {A}}$ was set as the identity matrix. By tuning the model uncertainty matrix, $\varvec{\mathrm {Q}}$, the time-varying characteristics of the model’s parameters can be observed [46]. The values of the matrix $\varvec{\mathrm {R}}$ represent the uncertainty of the measurements. In this case, the uncertainty is due to high-frequency noise introduced by the numerical calculation of angular velocities and accelerations.

## Data Availability

The datasets used and/or analyzed during the current study are available from the corresponding author on reasonable request.

## Abbreviations

GTO: Golgi tendon organ
KF: Kalman filter
LSM: Least squares method
RMSE: Root mean squared error
